# Testing the effectiveness of the Internet-based instrument PsyToolkit: A comparison between web-based (PsyToolkit) and lab-based (E-Prime 3.0) measurements of response choice and response time in a complex psycholinguistic task

**DOI:** 10.1371/journal.pone.0221802

**Published:** 2019-09-04

**Authors:** Jonathan Kim, Ute Gabriel, Pascal Gygax

**Affiliations:** 1 Department of Psychology, Norwegian University of Science and Technology, Trondheim, Norway; 2 Department of Psychology, University of Fribourg, Fribourg, Switzerland; Radboud Universiteit, NETHERLANDS

## Abstract

To test the effectiveness of the Internet-based instrument PsyToolkit for use with complex choice tasks, a replicability study was conducted wherein an existing psycholinguistic paradigm was utilised to compare results obtained through the Internet-based implementation of PsyToolkit with those obtained through the laboratory-based implementation of E-Prime 3.0. The results indicated that PsyToolkit is a viable method for conducting both general and psycholinguistic specific experiments that utilise complex response time tasks, with effects found to replicate for both response choice *and* response time.

## Introduction

The advent of the Internet opened new avenues of exploration for us as psychological researchers. Internet-based experimental instruments allow us to conduct experiments with demographically and culturally diverse samples, to recruit large subject pools in less time, to avoid organisational issues such as scheduling conflicts, to save costs related to laboratory space, equipment, personnel hours, and administration, and to increase our ability to conduct international experiments [1,2,3]. For these benefits to be worthwhile we must be able to trust Internet-based instruments to accurately record participants’ responses, in terms of both the actual responses as well as their intrinsic characteristics, such as response times. The current study investigates this particular issue by testing the replicability of the Internet-based implementation of PsyToolkit for use with paradigms requiring complex Choice Response Time (CRT) tasks.

It has been argued that, for instruments found to reliably record participants’ responses, Internet-based experimentation has three main advantages over laboratory-based experimentation [2]; increased generalisability, increased voluntariness, and increased ecological validity. Increased generalisability refers to participants being able to be recruited from much broader demographic and/or geographic backgrounds, meaning that the sample is more likely to be truly representative of society. Increased voluntariness refers to participants having fewer constraints on their decisions to participate and to continue to participate as, for example, there is no researcher whose presence might socially pressure a participant to continue. Further, responses may be more authentic when participants are more comfortable in their ability to stop the experiment [2]. Ecological validity is a measure of the level to which participant behaviour in an experiment resembles their behaviour in a naturalistic setting. The closer to reality an experiment can be, the higher the level of ecological validity the experiment is said to have, and the more we can be confident that the results obtained reflect the participant’s real-world behaviours. As an example, driving simulators attempt to simulate, to different degrees, the feeling of driving a real car. The closer the simulator is to the experience of naturalistically driving a car, the higher the level of ecological validity. As such, an experiment in which you sit inside an actual car, observe a scene projected on the wall in front and to the sides of you, and respond using the car’s steering wheel, accelerator, and break is likely to have a higher level of ecological validity than an experiment in which you sit in front of a computer screen, observe a scene shown on the screen, and respond using controllers shaped like a steering wheel, accelerator, and break, which in turn is likely to have a higher level of ecological validity than an experiment in which you sit in front of a computer screen, observe a scene shown on the screen, and respond by moving the mouse on the screen to control direction and speed. With reference to internet-based studies, it has been argued that the ability for participants to take part in experiments in environments (and using equipment) that they are familiar with, and the ability for participants to undertake experiments without the presence of a researcher in the room, lead to increased ecological validity [2].

The ability to undertake experiments in familiar environments, and with familiar equipment, has the potential to enhance ecological validity in at least two manners; increased familiarity and reduced cognitive load. Increased familiarity refers to the fact that participants can choose the time, place, and surroundings in which to undertake the experiment, ensuring that any effects found cannot be attributed to being in an unfamiliar setting [2]. Cognitive load refers to the amount of cognitive resources required, out of a limited pool, to fulfil the requirements of mentally demanding tasks [4]. In experimental terms, increasing levels of cognitive load are associated with increased reaction times, as participants have less cognitive resources available for undertaking experimental tasks. Unfamiliar environmental factors are known to increase cognitive load, as the level to which the brain actively monitors the environment is higher, which in turn reduces the cognitive resources available for other tasks. As such, the more familiar an individual is with their surroundings, the less cognitive resources are utilised in monitoring the environment, meaning that there are more cognitive resources available for focusing on the experimental task with which they are presented.

The lack of a researcher present has the potential to enhance ecological validity through reduced social desirability bias and reduced cognitive load. Social desirability bias refers to a cognitive bias in which individuals act to increase the level to which answers they give are in line with social norms in order to present themselves in the best possible light [4, 5]. The level to which this bias occurs is, among other factors, heightened in the presence of others [6]. As such, responses given in the absence of researchers are more likely indicative of how an individual truly feels about the subject, leading to higher ecological validity. Cognitive load is also reduced in the absence of a researcher, as the presence of others when undertaking a task divides attention, at least to some degree, between the experimental task and anyone else present [7, 8].

While increasing ecological validity is an important factor for experimental design, laboratory-based experiments also have advantages over internet-based experiments. Firstly, laboratory-based experiments have a higher range of possible research approaches. This is primarily due to equipment requirements. It is not reasonable, for example, to expect participants recruited from the general populace to all own eye tracking equipment; as such, it is more logical to undertake experiments in which eye tracking is included in laboratory conditions. Further, hardware and software related issues have historically introduced a high level of error noise into results obtained through internet-based instruments compared to those obtained through laboratory-based instrumnets, primarily observable as response time noise. A wide variety of factors can affect response time recording, such as hardware timing features, device driver issues and interactions, script errors, operating system variability, interactions with other software, tools to construct the paradigm, interactions with other hardware, and configuration of settings and levels [3,9]. In laboratory-based experiments these sources of noise are less likely to affect the final results of the experiment, as all participants undertake the experiment with the same hardware, software, device drivers, operating system, and system configuration. In internet-based experiments, however, there are large potential differences in these elements between participants’ computers, which can lead to a higher level of noise within the results obtained. Further, responses given via the internet are also affected by the amount of time it takes for the website hosting the experiment to successfully send an image to the participants’ computer, and then, after responding, by the amount of time it takes for the response to be sent from the participants’ computer to the website hosting the experiment [10]. As high noise levels can obscure small effects and give the illusion of heterogeneous responses, care must be taken when analysing results obtained through internet-based instruments to ensure that an increase in heterogeneous responses are due to ecological validity improving rather than noise level increasing. However, technology continues to evolve, and recent advances in the design of internet-based experimental tools–such as has occurred with PsyToolkit, the instrument we present next–may have significantly reduced error noise compared to older internet-based instruments, even to the point of bringing them fully in line with laboratory-based instruments. As such, for instruments with which there is minimal Internet-related noise, if ecological validity was indeed increased we could (for example) expect participants to respond to items in a less self-monitored and/or socially accepted manner, with participants displaying wider response choice variability and overall faster response times.

PsyToolkit is an open-access psychological instrument developed to allow researchers, including student researchers, to easily program and run experimental psychological experiments and surveys for both laboratory and Internet settings [11,12]. Two versions of PsyToolkit are available; a laboratory-based version that runs on Linux, and an Internet-based version that is Javascript based and can run on modern browsers without participants needing to download any programs. The Internet-based version of the instrument is specifically aimed at addressing financial and technical limitations commonly faced by students, as it is free software that has specifically been designed for running online questionnaires, Simple Response Time (SRT) tasks, and Choice Response Time tasks (CRT) [12]. A SRT is an experimental task in which a single stimulus, and only that stimulus, is presented repeatedly at the same on-screen location, with participants tasked with responding to every presentation of the stimulus in the exact same manner and quickly as possible [13]. An example of this is participants being instructed to watch an LED and to press a specific button as quickly as possible whenever the LED lights up. A CRT is an experimental task in which instead multiple stimuli are shown, and/or stimuli are presented on different areas of the screen, and the participant is tasked with responding in different manners depending on the nature of each presentation (e.g., Zajdel and Nowak [13]). An example of this is participants being instructed to look at a screen on which letters will appear, with the task of pressing the corresponding letter on a keyboard. CRTs can also differ in complexity. Simple CRTs, such as in the above example, require participants to recognise the stimuli and respond accordingly. More complex CRTs require participants to also make judgements about the nature of the stimuli.

In the present experiment, participants were instructed to look at a screen on which first names paired with role nouns appeared, with the task of pressing one of two buttons depending on whether they believed that it made logical sense for someone with the name shown to hold the role shown. Stoet [12] states that PsyToolkit is designed for a teaching environment, with minimal technical barriers and free web-based hosting of their studies. A library of existing psychological scales and experiments is available for students to examine and adapt, and extensive online documentation and tutorials are available to assist if students face any issues. Further, Stoet [12] states that PsyToolkit is designed to allow for students to randomise item order in both questionnaires and in cognitive experiments, to allow for a convenient way of scoring, and to give feedback to participants about their test scores; options not available in all Internet-based instruments. All users of the Internet-based version must register an account to be able to create experiments, but accounts are free. Randomisation is possible in both the survey and the experiment, and partial randomisation is also possible for if one wishes for only certain portions of the survey and/or experiment to be randomised. Further, alternate versions of the experiment can be created, with participants randomly assigned between versions. In terms of reliability, Stoet [14] states that both the Internet and Linux versions of PsyToolkit can reliably measure small effects of less than 50ms, with the Linux version being more precise. However, to our knowledge, currently no research has been published examining the replicability of the Internet-based version of PsyToolkit.

As PsyToolkit is intended to be a student-focused instrument, and many universities do not set up experimental computers with Linux for their students, it was decided to compare results obtained through the Internet-based implementation of PsyToolkit to results obtained through E-Prime 3.0 in a laboratory setting. E-Prime was chosen as it is a commonly used psychological research tool in university settings, including in teaching environments, and, like PsyToolkit, it has a low barrier to entry and has an experiment library. Further, Stoet [14] states that the Linux-based version of PsyToolkit is on par with E-Prime, so, while there is likely to be noise due to differences in software, this is expected to be minimal.

While the replicability of PsyToolkit has not been examined, the replicability of other Internet-based instruments has been tested through CRT tasks (e.g., Reimers and Stewart [15]; Schubert, Murteira, Collins, & Lopes [16]). Reimers and Stewart [15] used a CRT task to test the replicability of an experiment in the Internet-based version of Adobe Flash compared to the same experiment in a laboratory-based version of Adobe Flash, with the same experiment coded in C used as a baseline. Participants were shown green and red rectangles and were required to press buttons corresponding to the colour of the rectangle on the screen. They found that, compared to the baseline, (a) response times of the laboratory-based version of Flash were 10ms longer, (b) response times of the Internet-based version of Flash were 30-40ms longer, and (c) there were no significant differences in Response Time standard errors across conditions. Schubert et al. [16] used both SRT and CRT experiments in a study testing the replicability of ScriptingRT to Flash. Six experiments were conducted over the course of their study. The first three studies used SRT tasks but were automated to test specific aspects of ScriptingRT. The last three studies used CRT tasks, specifically a version of the Stroop task, where participants were presented with either the words “red” or “blue”, or a neutral letter string, in either red or blue on a white background. Participants were instructed to press keys corresponding to the colour of the word or neutral letter string shown. Experiment 4 tested the Internet-based version of ScriptingRT by itself, while Experiment 5 compared ScriptingRT to the same experiment coded in DMDX (a laboratory-based instrument [17]) with participants undertaking both tasks on the same computer, and Experiment 6 compared ScriptingRT to Inquisit Web Edition, both running via the Internet. In Experiment 5, the experiment of most interest to the present experiment as it compares an Internet-based implementation of the instrument to a laboratory-based one, Schubert et al. [16] found that the size of the Stroop effect was not affected by which software was used.

Historically, psycholinguistic research has not relied upon Internet based testing, as it often relies upon small differences in response times in CRT tasks to detect effects [18] and is strongly affected by response time noise. Recent research (e.g., Enochson & Culbertson [18]) has found that some modern Internet-based instruments are reliably able to test these small differences, meaning that modern psycholinguistic research may safely utilise Internet based tools that have been properly validated. Some researchers have suggested that PsyToolkit may be a delicate enough tool for psycholinguistic experimentation (e.g., Sampaio [19]). An opportunity arises therefore to test both general replicability and psycholinguistic specific replicability of PsyToolkit through a psycholinguistic experimental paradigm.

The present study was designed to compare responses and Response Times measured by the Internet-based implementation of PsyToolkit with those measured by the laboratory-based implementation of E-Prime 3.0 using a complex CRT task composed of an existing and published psycholinguistic paradigm (i.e., Gygax & Gabriel [20]) to test replicability between the Internet-based implementation of PsyToolkit (Version 2.4.3) and E-Prime (Version 3.0.3.31). The paradigm uses a between-subjects two-alternative forced choice design, with a CRT task in which participants are shown pairs of terms (in the present experiment a first name and a role noun; e.g., ‘Kate–Chefs’) and are then required to, as quickly as possible, make a judgement as to whether the pairing makes logical sense (i.e., could someone named Kate be a member of a group of chefs). Experimental item pairings were composed of first names paired with professional roles that vary in gender stereotypicality. As logically any individual can hold any professional role, filler item pairings were included to prevent participants of developing a strategy of always answering positively to all roles seen. The filler items were first names paired with gender-marked kinship terms, with both congruent (e.g., ‘Kate–Mothers’) and incongruent (e.g., ‘Kate–Fathers’) pairings shown to prevent participants from developing a strategy of answering positively to professional roles and negatively to familial roles.

The paradigm we utilise is more complex than those used by Reimers and Stewart [15] and by Schubert et al. [16], as the paradigm used in the current study requires participants to make subjective judgements of the items presented before responding, while the paradigms used by Reimers and Stewart [15] and Schubert et al. [16] required that participants responded based on the colour, an objective quality, of the items presented to them. One can therefore expect that overall response times will be longer for this study than those found by Reimers and Stewart [15] and Schubert et al. [16], and, compared to Reimers and Stewart [15], it is likely that Response Time standard errors will be larger. Further, if the results indicate that there is a high level of replicability between PsyToolkit and E-Prime, then it may be possible to determine whether the results offer any support for the concept of increased ecological validity in Internet-based experiments. If the results obtained in PsyToolkit do have a higher level of environmental validity than the results obtained in E-Prime, we would expect that participants who undertake the PsyToolkit version of the experiment would be more likely to respond negatively, and would overall respond more quickly (i.e., more spontaneously), than those who undertake the E-Prime version of the experiment.

It is worth noting that Norwegian is considered a semi-gendered language. This is because some, but not all, nouns have associated gender markers. Specifically, only nouns that refer to living beings, especially humans, are gendered in Norwegian. Further, the majority of role nouns in the plural form are the same as the masculine-specific singular form. This is due in part to a linguistic policy of gender neutralisation [21], under which the masculine grammatically marked form of role nouns are actively encouraged to become the main linguistic device to refer to the majority of roles [22].

## Method

### Participants

A total of 81 participants took part in this study (39 [18 female, 20 male, 1 nonbinary] through PsyToolkit, 42 [20 female] through E-Prime). Across both versions of the experiment participants were between 19 and 31 years old (*M* = 23.4; *SD* = 2.3), were self-reported Norwegian first language speakers, and were currently studying at NTNU, Norway. Participants in both PsyToolkit (Version Web) and the E-Prime (Version Lab) were recruited through posters and flyers placed around the Dragvoll campus at NTNU, and through direct recruitment (i.e., the researchers involved approaching people directly and asking whether they would be willing to take part in the experiment). Those who responded to the advertisements or to the direct recruitment were then either asked to undertake Version Lab at the Dragvoll campus of NTNU or were sent a link to undertake Version Web. Recruitment into both versions occurred concurrently. All participants were compensated through coffee vouchers. Informed consent was obtained from all participants prior to the experiment. This study received approval from the Norwegian Centre for Research Data.

### Materials and research design

A two-alternative forced choice design was used for both versions of the experiment. All experimental elements were translated into Norwegian. Participants gave informed consent, answered questions on age, gender, and handedness, and stated whether they were currently enrolled university students, before experimental onset. For Version Web this was done through a form on the website hosting the experiment, while for Version Lab this was done in hard copy.

Participants were presented with pairs of terms composed of a first name (e.g., Daniel) and a role noun in the plural form (e.g., Astronauts). Participants were then required to indicate, as quickly as possible, whether someone named [name] could be a member of the group of [noun]. These pairings were always presented in the form ‘[name]–[noun]’ (e.g., Daniel–Astronauts), with presentation order randomised by participant. Participants in both versions of the experiment responded via a keyboard, and were instructed to press ‘e’ if they did not agree that the individual could be a member of the group indicated, or ‘i’ if they did agree. After each answer was given, the pairing was replaced with a fixation cross of 100ms, after which the next pairing was displayed. The lack of a response within 5000ms was recorded as a non-response, after which the experiment would continue. Participants undertook a five-item training phase before undertaking the main experimental phase. Both versions of the experiment took between 20 and 30 minutes to complete.

#### Stimuli

The stimuli were composed of six first names paired with 36 role nouns and 36 filler items. In total, participants were presented with 360 noun-name pairings, composed of 216 experimental pairings and 144 filler pairings.

The 36 role nouns (12 female stereotyped roles, 12 male stereotyped roles, and 12 non-stereotyped roles; Tables 1–3) were selected based on Misersky et al. [23]. Misersky et al. produced stereotypicality rankings between 0 and 1, with 0 representing male stereotyped roles, 0.5 representing non-stereotyped roles, and 1 representing female stereotyped roles. For this study, the masculine roles selected had a mean rating of .20 (SD = .03), while the feminine roles had a mean rating of .81 (SD = .04), and the non-stereotyped roles had a mean rating of .53 (SD = .06).

**Table 1:** Stereotypicality score for feminine experimental role nouns as determined from the findings of Misersky et al.

Role noun	English translation	Score	SD
Manikyrister	Manicurists	.88	.08
Bryllupsplanleggere	Wedding planners	.85	.10
Kosmetikere	Beauticians	.85	.10
Eksotiske dansere	Exotic dancers	.83	.10
Prostituerte	Prostitutes	.83	.13
Strippere	Strippers	.81	.18
Fødselshjelpere	Birth attendants	.80	.13
Frisører	Hairdressers	.79	.11
Barnevakter	Childminders	.78	.13
Groupier	Groupies	.77	.17
Synske	Clairvoyants	.76	.12
Sekretærer	Secretaries	.75	.10
*Mean*		.81	.12

**Table 2:** Stereotypicality score for masculine experimental role nouns as determined from the findings of Misersky et al.

Role noun	English translation	Score	SD
Fabrikkbestyrere	Factory managers	.25	.13
Fyrvoktere	Lighthouse keepers	.24	.15
Guvernører	Governors	.23	.12
Datateknikere	Computer technicians	.23	.09
Skogsforvaltere	Forest rangers	.22	.14
Trommeslagere	Drummers	.21	.11
Astronauter	Astronauts	.20	.12
Brytere	Wrestlers	.20	.18
Søppeltømmere	Rubbish collectors	.17	.11
Taktekkere	Roofers	.17	.14
Kranførere	Crane operators	.15	.10
Soldater	Soldiers	.15	.11
*Mean*		.20	.13

**Table 3:** Stereotypicality score for non-stereotyped experimental role nouns as determined from the findings of Misersky et al.

Role noun	English translation	Score	SD
Fysioterapeuter	Physiotherapists	.60	.11
Miljøaktivister	Environmentalists	.60	.12
Fiolinister	Violinists	.59	.14
Arkivarer	Archivists	.57	.19
Meteorologer	Meteorologists	.55	.19
Akrobater	Acrobats	.53	.13
Kunstnere	Artists	.53	.11
Fagforeningsmedlemmer	Trade unionists	.51	.10
Fotografer	Photographers	.51	.13
Biologer	Biologists	.46	.16
Oceanografer	Oceanographers	.45	.14
Idrettsutøvere	Athletes	.42	.11
*Mean*		.53	.14

Three female (Ida, Nina, Sandra) and three male (Espen, Geir, Robert) names were used to maintain gender balance. These were selected based on the findings of Öttl [24], who tested typicality of names through a response time experiment. In Öttl’s experiment participants were presented with names and were instructed to press a button marked ‘female’ if they thought the name was female, and ‘male’ if they thought the name was male. The names used were taken from Statistics Norway, and were selected to represent the most frequent Norwegian names among people born between 1976 and 1996. Lower response times were interpreted as indicating a higher level of gender typicality associated with that name. The typicality of the names selected for the current study was balanced by gender (Table 4). Each name was paired with all role nouns, for a total of 216 experimental pairings.

**Table 4:** Typicality of female and male first names as indicated by response time results from the findings of Öttl.

First Name Gender	Name	Mean Response Time
Male	Espen	566ms
	Geir	574ms
	Robert	583ms
Female	Ida	584ms
	Nina	565ms
	Sandra	573ms

The 36 filler items were gender-marked kinship terms (e.g., Father, Sister; 18 female gender marked, 18 male gender marked) that were selected to prevent participants developing a strategy of always answering positively. Kinship terms were paired with both incongruent and congruent names so that participants would be unlikely to adopt a strategy of responding positively to all items, but would also be unlikely to adopt a strategy of responding positively to professional roles but negatively to kinship terms. Each name was paired with all of the incongruent filler items, for a total of 108 first name–incongruent filler item pairings, and was paired with six of the congruent filler items, for a total of 36 first name–congruent filler item pairings.

#### Procedure for Version Web

Participants undertook this version of the experiment on their home computers, for which we do not have the specifications. The experiment was run through the PsyToolkit website. Before starting the survey, participants were required to give informed consent through a check-box on the website. Failure to check this box meant that the survey would not begin. During the first part of the survey, participants answered the demographic questions stated above. After this, a black box was shown on the screen, and participants were instructed to click a button underneath it to start the experiment when they were ready. When this button was pressed, the black box expanded to full-screen mode, and the experiment began. Responses were only saved by PsyToolkit if participants completed the survey and experiment in entirety, with all survey questions needing to be answered before participants could move on. After completing the experiment, participants were presented with a code and were instructed to email the code to the researchers to arrange a time to receive their compensation. As emails constitute identifying information, the emails were deleted after participants received their compensation. PsyToolkit created two files per participant. The first contained information relating to when they started and ended the experiment, their IP address, and their responses to the demographic questions. The second contained their responses to each of the experimental pairings.

#### Procedure for Version Lab

Participants undertook this version of the experiment in a laboratory setting in the Psychology Department at NTNU. Before starting the experiment, participants were required to give informed consent, and then to answer demographic questions, through hard-copy forms. After this, participants undertook the experiment. This was presented to them on a screen (1920 x 1080), which was attached to an air-gated Dell Latitude E5470 laptop with an Intel core i7-6820HQ CPU and 16gb RAM, running Windows 10 Education in 64-bit, with a screen refresh rate of 60Hz. The laptop sat facing the researcher, while the screen sat facing the participant. The participant was seated directly opposite the researcher, so that they faced each other but direct line of sight during the experiment was blocked by the screen. The display was mirrored between the laptop and the connected screen. A USB keyboard was attached to the laptop, and placed in front of the participant. After the participant had given informed consent and filled in the demographics questionnaire, the researcher present initiated the experiment. Participants received compensation directly after completing the experiment. E-Prime created two files per participant. These both contained the participant’s responses to the experimental pairings, with one being in the .edat3 format, and the other in the .txt format.

### Data preparation

For the analysis, demographic information for all participants in each version of the study was compiled into two .txt files, while the experimental data was kept in its uncollated raw form as .txt files for each participant. As IP addresses are identifying information, in order to anonymise the data they were removed from the demographic information files and deleted prior to data analysis.

Prior to data analysis, both item-by-participant deselection and by-participant data screening were used. Item-by-participant deselection was conducted based on response times. In keeping with standard procedures, such as in Schubert et al. [16], responses faster than 300ms or not occurring within 5000ms were removed from the data. This represented 0.75% of the data. By-participant data screening was composed of removing participants who (a) were outside of our target demographic group (native Norwegian speaking university students aged between 18 and 35), and (b) removing participants who were found to have an error rate at or above 50%. Error rate by participant was calculated based on the percentage of incorrect answers to all filler items, with the assumption that the correct answer for congruent name–filler item pairings is ‘yes’ and for incongruent name–filler item pairings is ‘no’. One participant was removed for not being a native Norwegian speaker, and seven were removed because their error rate was above 50%. All of the participants deselected in this manner took part in Version Lab. The remaining 37 participants who completed Version Web (18 female, 18 male, 1 nonbinary) and 36 participants who completed Version Lab (18 female, 18 male, 0 nonbinary) were used for analysis (*N* = 72). After deselection, mean participant age was 23.4 (SD = 2.4).

Mean error rate across the study and by version of the experiment was calculated post data screening and deselection. Mean error rate across the study was 11.56%. Mean error rate for Version Web was 10.76%, while mean error rate for Version Lab was 12.38%.

The results were examined through two forms of linear mixed-effects modelling. First, participants’ responses (yes/no) were analysed, and second, response times for positive responses were analysed (as in Gygax, Gabriel, Lévy, Pool, Grivel, & Pedrazzini [25]), both within and between versions of the experiment. Participants’ yes/no responses were modelled through *generalised* linear mixed-effect regression, while participants’ response times for positive responses were modelled through linear mixed-effect regression. Analysis was conducted through the *glmer* and *lmer* functions of the lme4 package (Version 1.1–12; Bates et al. [26]) in R (version 3.3.3). Initial models were defined for both analyses, composed of all experimental factors (Version [Version Web vs. Version Lab], Name Gender [female vs. male], and Stereotype [female vs. male vs. non-stereotyped roles]), their 2-way and 3-way interactions, and random intercepts (Participants, Role Noun, Researcher, and First Name). Researcher refers to which researcher, if any, was present while participants undertook the experiment. All participants who undertook Version Lab did so in the presence of a researcher, while all participants who undertook Version Web did without a researcher present. The models also included fixed effects of Participant Gender, Handedness, Trial Number and Character Count (i.e., how many characters [specifically letters, symbols, and spaces] were in each name–noun pairing). In keeping with Baayen [27] and Baayen and Milin [28], refinement to find the model of best fit occurred through back-fitting the fixed effects structure, forward-fitting the random effects structure (by-participant random slopes for the experimental variables, trial number, and number of characters), then re-back-fitting the fixed effect structure. This was done automatically through the *bfFixefLMER_F*, *ffRanefLMER*, and *fitLMER*.*fnc* functions of the lme4 package. Post-hoc analysis for main effects and interaction effects was done through the *effects()* function of the effects package (version 4.1–0, Fox [29]).

Effect size estimates in linear mixed-effects modelling are complicated to determine, with a large variety of practices being utilised in research [30]. To best fit our data, we have selected two methods which address local effect sizes. For this purpose, we utilise the definition of local effect sizes as the effect of individual fixed effect variables on the dependent variable [30]. In line with previous research [31, 32], estimation of local effect sizes is done through partial omega squared ($\omega_{p}^{2}$), obtained through the *omega_sq()* function of the *sjstats* package (Version 0.17.5, Lüdecke [33]). Further, in keeping with previous research [32], we present the slopes of the reported effects for each individual level, along with their 95% confidence intervals. The estimation of the slope for the effects was done through the *summary()* function in R, while 95% confidence intervals were calculated through the equation [CI = slope estimate ± (Critical value * Standard error of the slope coefficient)].

## Results

### Response

The AIC value for the initial model was 4885. Version (Web vs. Lab) was automatically removed from the model of best fit during backfitting. However, as this study aims at exploring the Version’s impact, Version was nevertheless kept in the final model. The final model for Response contained random intercepts by Role Noun, First Name, Researcher, and Participant, as well as random slopes of Stereotype by Participant, Name Gender by Participant, and Trial Number by Participant. The AIC value for the final model was 4660. Trial Number was found to have a small significant effect on the model (Wald Chi^2^ = 20.41, *p* < 0.001, $\omega_{p}^{2}=0.005$), with participants increasingly likely to respond positively over time. There was a small yet significant main effect of Name Gender (Wald Chi^2^ = 36.92, *p* < 0.001, $\omega_{p}^{2}=0.001$), which was qualified by a medium sized and significant two-way interaction between Stereotype and Name Gender (Wald Chi^2^ = 564.08, *p* < 0.001, $\omega_{p}^{2}=0.114$). There was no significant main effect of Version (Wald Chi^2^ = 0.01, *p* = 0.930, $\omega_{p}^{2}=0.000$) or of Stereotypicality (Wald Chi^2^ = 1.76, *p* = 0.410, $\omega_{p}^{2}=0.000$). No significant two-way interactions were found between Stereotype and Version (Wald Chi^2^ = 0.57, *p* = 0.750, $\omega_{p}^{2}=0.000$), or between Name gender and Version (Wald Chi^2^ < 0.001, *p* = 0.990, $\omega_{p}^{2}=0.000$). No significant three-way interaction was found between Stereotype, Name Gender, and Version (Wald Chi^2^ = 4.07, *p* = 0.13, $\omega_{p}^{2}=0$). Estimates of the slope sizes and confidence intervals for the final model can be found in Table 5.

**Table 5:** Effect sizes for the fixed effects in the model ‘effect of Version (web-based vs. Laboratory Based) on positive responses. Table shows the estimated effect size and 95% confidence intervals. Intercept included Masculine Roles, Female Names, and Version Web as contrast levels.

Fixed effect	Effect size	Lower Bound	Upper Bound
Intercept	4.856	2.812	6.899
Trial Number	0.006	0.004	0.008
Feminine Roles	0.281	-0.413	0.975
Non-Stereotyped Roles	-0.322	-0.960	0.316
Male Names	0.029	-0.172	0.230
Version Lab	0.201	-1.741	2.143
Feminine Roles: Male Names	-1.715	-1.871	-1.559
Non-stereotyped Roles: Male Names	0.288	0.128	0.448
Feminine Roles: Version Lab	0.112	-0.211	0.435
Non-stereotyped Roles: Version Lab	-0.137	-0.420	0.146
Male Names: Version Lab	-0.007	-0.216	0.202
Feminine Roles: Male Names: Version Lab	-0.142	-0.287	0.003
Non-stereotyped Roles: Male Names: Version Lab	0.048	-0.110	0.206

**Fig 1:**
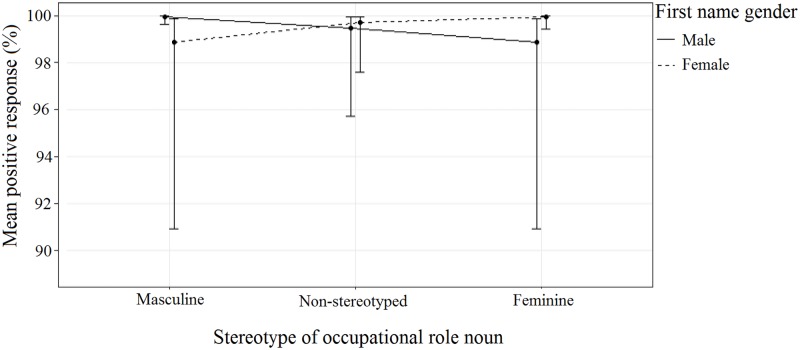
The effect of Stereotype and Name Gender on response. Error bars indicate the 95% confidence interval.

The interaction between Stereotype and Name Gender (Fig 1, Table 6) indicated no significant differences between conditions, but that participants were, on average, more likely to respond positively to congruent pairings (i.e., pairings where the gender of the first name matched the stereotype of the role noun) than to the incongruent pairings (i.e., pairings where the gender of the first name does not match the stereotype of the role noun) for both male and female names. Participants also tended to respond more positively to names when paired with non-gender-stereotyped roles than with incongruent roles for both female and male names, and tended to respond more positively to non-gender-stereotyped roles when paired with female names compared to male names.

**Table 6:** The effect of the two-way interaction between Stereotype and Name Gender on response. Table shows mean positive response (%) and 95% confidence interval for each name/role pairing, rounded to the nearest full percentage.

Stereotype	Name Gender	Mean Response	Lower Bound	Upper Bound
Feminine	Female	100	99	100
	Male	99	91	100
Non-Stereotyped	Female	100	98	100
	Male	99	96	100
Masculine	Female	99	91	100
	Male	100	100	100

**Fig 2:**
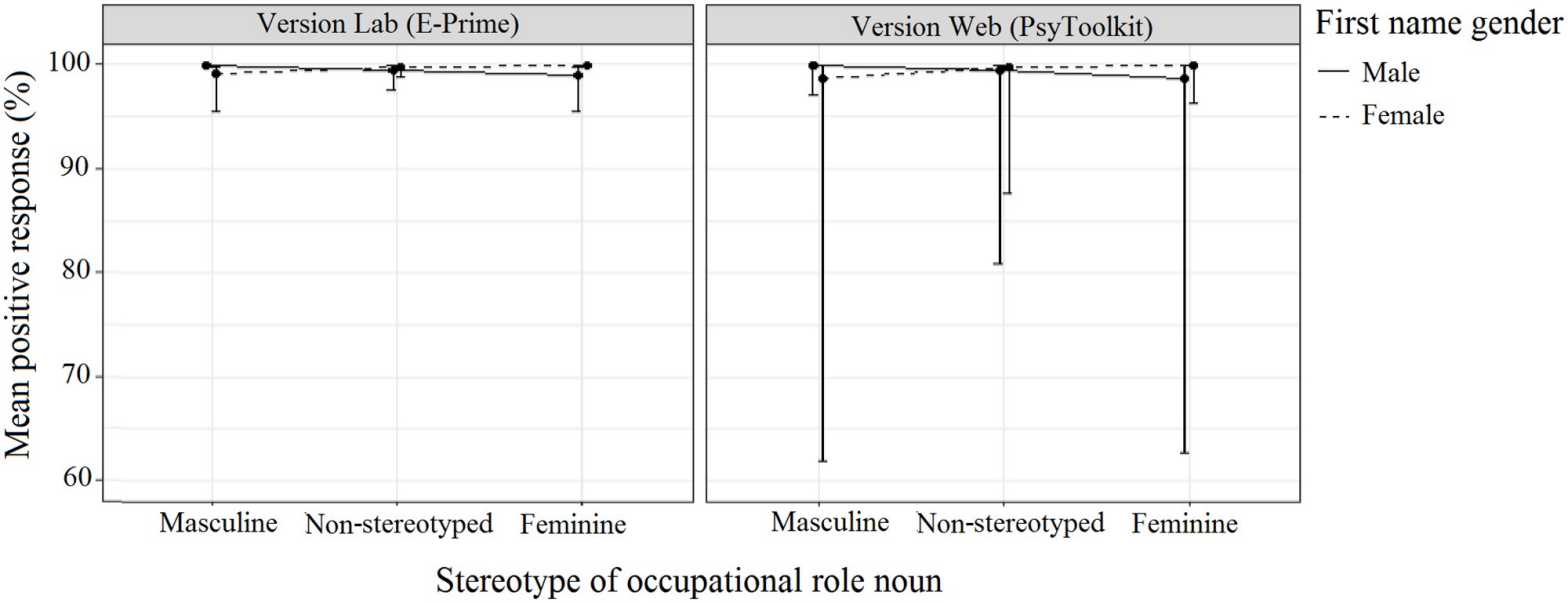
The effect of Version, Stereotype, and Name Gender on response. Error bars indicate the 95% confidence interval.

As it was of importance to this study, we will still discuss the non-significant interaction between Stereotype, Name Gender, and Version (Table 7, Fig 2). No significant differences were observed between conditions, and a visual scan of Fig 2 indicates that participants across both versions of the experiment tended to respond more positively to congruent pairings compared to incongruent pairings. Participants who responded to Version Web showed more variation in the responses they gave, resulting in much lower Lower Bound values compared to Version Lab, with the largest differences observed for the incongruent role noun pairings.

**Table 7:** The effect of the three-way interaction between Version, Stereotype, and Name Gender on response. Table shows mean positive response (%) and 95% confidence interval for each name/role pairing, rounded to the nearest full percentage.

Version	Stereotype	Name Gender	Mean Response	Lower Bound	Upper Bound
Version Web (PsyToolkit)	Feminine	Female	100	96	100
		Male	99	63	100
	Non-Stereotyped	Female	100	88	100
		Male	99	81	100
	Masculine	Female	99	62	100
		Male	100	97	100
Version Lab (E-Prime)	Feminine	Female	100	100	100
		Male	99	95	100
	Non-Stereotyped	Female	100	99	100
		Male	99	98	100
	Masculine	Female	99	96	100
		Male	100	100	100

As Version was automatically removed from the model of best fit, Bayes factors were calculated to examine whether there was support for accepting or rejecting the null hypothesis (i.e., that there was no difference between Version Web and Version Lab). This was done with both the *BF_BIC* function of the *lme4* package [26]. The comparison models used for this analysis were the final model (stated above) compared to the model of best fit. The model of best fit was identical to the final model aside from the removal of the main effect and interaction effects of Version. The results indicated a Bayes factor of > 0.001, indicating that we can confidently accept the null hypothesis for Response.

### Response time

The REML criterion at convergence for the initial model was 212699. Two- and three-way effects involving Version (i.e., Web vs. Lab) were automatically removed from the model of best fit during backfitting, although, unlike with Response, the main effect of Version was kept in the model of best fit. Aswith Response, the two- and three-way effects involving Version were kept in the final model due to their importance in this study. In order to correct for outlier responses, the final model excluded responses that were more than 2.5 standard deviations from the mean. The final model for Response Time contained random intercepts by Role Noun, First Name, Researcher, and Participant, as well as random slopes of Character Count by Participant and Name Gender by Participant. The REML criterion at convergence for the final model was 199660. Trial Number was found to have a large and significant effect on the model, *F*(1, 13664) = 156.19, *p* < 0.001, $\omega_{p}^{2}=0.105$, with participants responding increasingly quickly over the length of the experiment. Character Count was also found to have a small yet significant effect on the model, *F*(1, 13664) = 29.10, *p* < 0.001, $\omega_{p}^{2}=0.002$, with participants responding increasingly slower as character count increased. A small but significant main effect of Stereotype, *F*(1, 13664) = 4.56, *p* = 0.01, $\omega_{p}^{2}=0.001$ was found, as well as a very small but significant main effect of Name Gender, *F*(1, 13664) = 4.90, *p* = 0.03, $\omega_{p}^{2}<0.001$, which were qualified by a small yet significant two-way interaction between Stereotype and Name Gender, *F*(1, 13664) = 20.87, *p* < 0.001, $\omega_{p}^{2}=0.003$. A significant main effect of Study was also found, *F*(1, 13664) = 28.91, *p* < 0.001, $\omega_{p}^{2}=0.002$, but no significant two-way interactions were found between Stereotype and Version, *F*(1, 13664) = 0.43, *p* = 0.653, $\omega_{p}^{2}<0.001$, or between Name Gender and Version, *F*(1, 13664) = 0.36, *p* = 0.547, $\omega_{p}^{2}<0.001$, and no significant three-way interaction was found between Stereotype, Name Gender, and Version, *F*(1, 13664) = 0.14, *p* = 0.867, $\omega_{p}^{2}<0.001$. Estimates of the slope sizes and confidence intervals for the final model can be found in Table 8.

**Table 8:** Effect sizes for the fixed effects in the model ‘effect of Version (web-based vs. Laboratory Based) on positive responses. Table shows the estimated effect size and 95% confidence intervals. Intercept included Masculine Roles, Female Names, and Version Web as contrast levels.

Fixed effect	Effect size	Lower Bound	Upper Bound
Intercept	6.993	6.859	7.127
Trial Number	-0.001	-0.001	-0.001
Feminine Roles	0.027	-0.002	0.055
Non-Stereotyped Roles	-0.027	-0.055	0.000
Male Names	-0.011	-0.031	0.008
Version Lab	-0.077	-0.149	-0.005
Number of Characters	0.009	0.003	0.014
Feminine Roles: Male Names	-0.026	-0.033	-0.018
Non-stereotyped Roles: Male Names	0.005	-0.002	0.013
Feminine Roles: Version Lab	-0.002	-0.017	0.013
Non-stereotyped Roles: Version Lab	-0.003	-0.016	0.010
Male Names: Version Lab	0.003	-0.008	0.014
Feminine Roles: Male Names: Version Lab	0.002	-0.006	0.009
Non-stereotyped Roles: Male Names: Version Lab	0.001	-0.007	0.008

**Fig 3:**
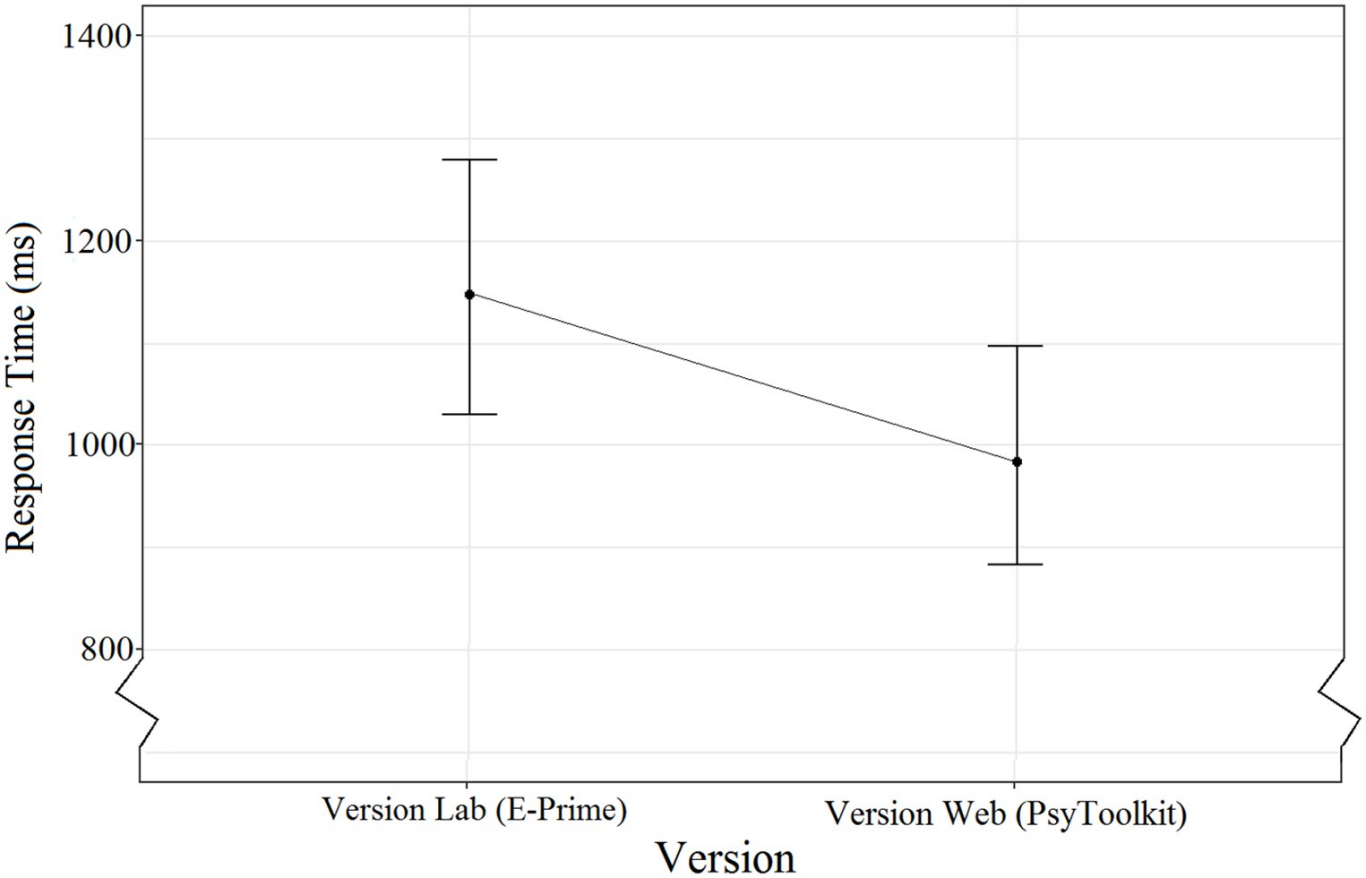
The main effect of Version on response time. Error bars indicate the 95% confidence interval.

The main effect of Version (Fig 3) indicated no significant differences between conditions, but that participants who responded to Version Web (mean response time = 985ms) responded faster on average than participants who responded to Version Lab (mean response time = 1148ms; mean difference = 163ms, 95%CI [-67ms to 396ms]).

**Fig 4:**
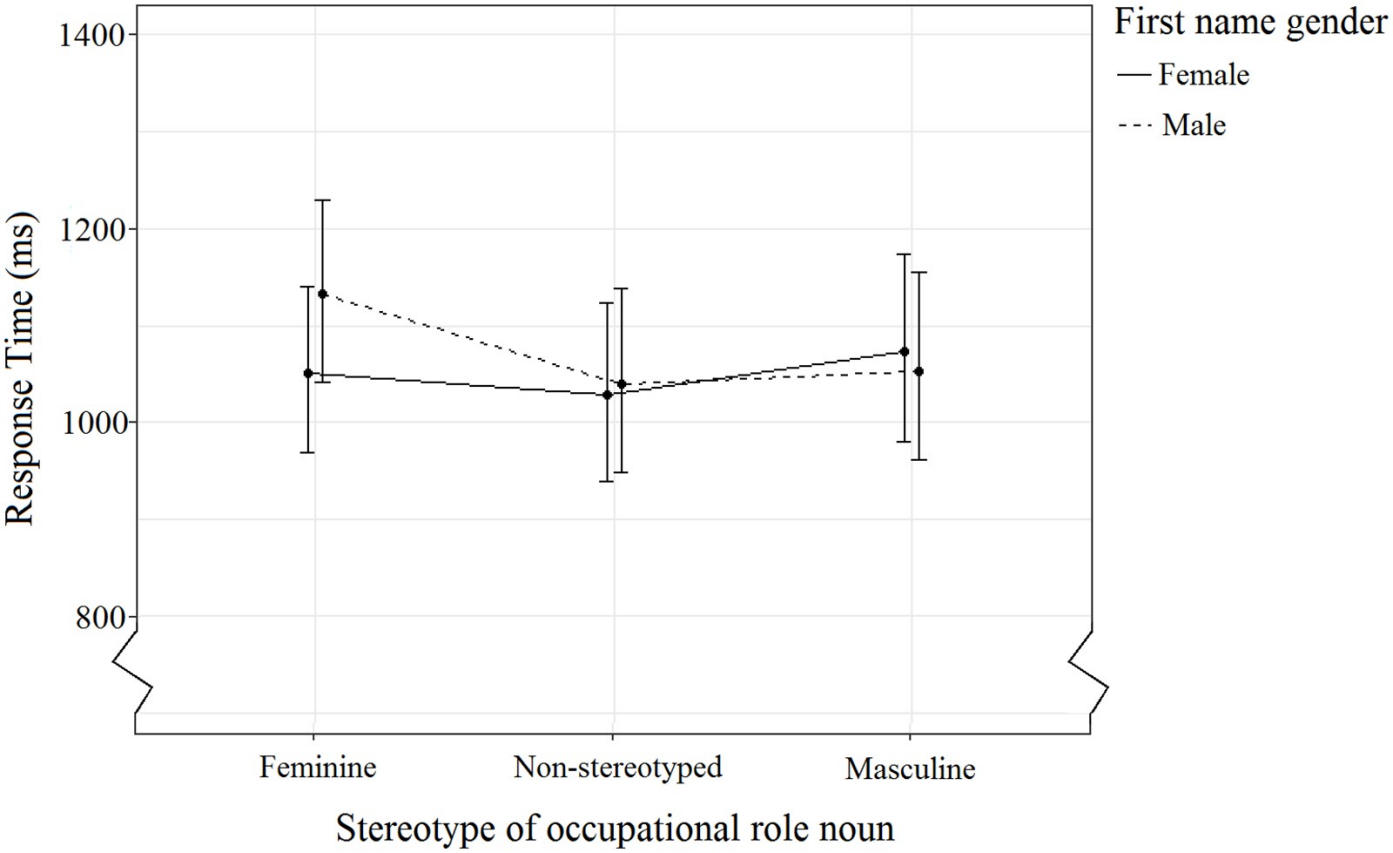
The effect of Stereotype and Name Gender on response time. Error bars indicate the 95% confidence interval.

The interaction between Stereotype and Name Gender (Fig 4, Table 9) indicated no significant differences between conditions, but that participants, on average, responded more quickly to the congruent roles compared to incongruent roles for both female and male names. Participants also tended to be slower to answer positively to male names paired with feminine stereotyped roles than to female names paired with masculine stereotyped roles.

**Table 9:** The effect of the two-way interaction between Stereotype and Name Gender on response time. Table shows mean response time (ms), SD, and 95% confidence interval for each name/role pairing.

Stereotype	Name Gender	Mean Response	SE	Lower Bound	Upper Bound
Feminine	Female	1052	44	970	1141
	Male	1132	48	1041	1230
Non-Stereotyped	Female	1028	47	940	1124
	Male	1040	48	949	1139
Masculine	Female	1073	49	980	1174
	Male	1053	49	961	1155

**Fig 5:**
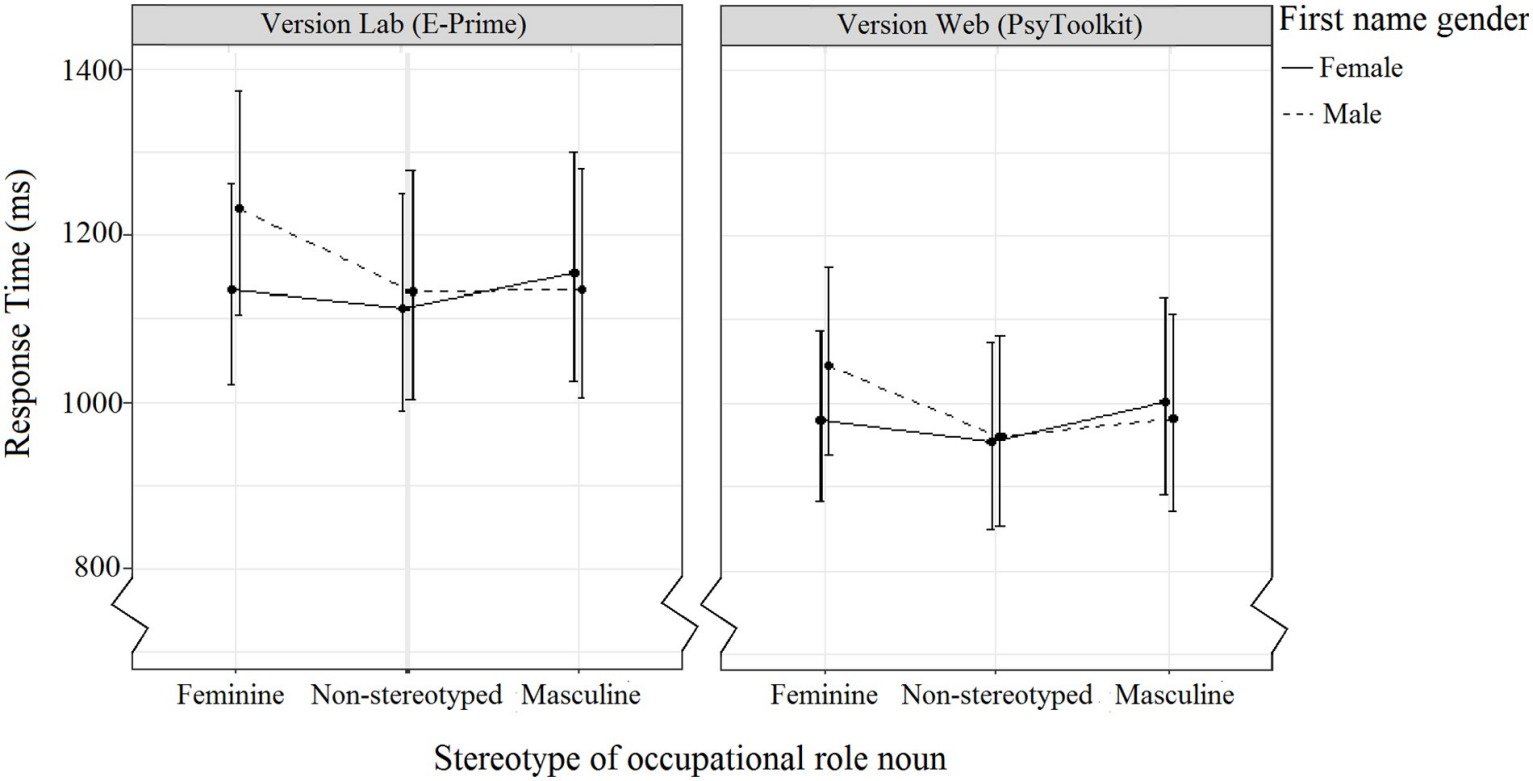
The effect of Version, Stereotype, and Name Gender on response time. Error bars indicate the 95% confidence interval.

Again, as it was of importance to this study, we examine still the non-significant interaction between Stereotype, Name Gender, and Version (Table 10, Fig 5). No significant differences between conditions, but there was an overall tendency for participants in Version Web to respond faster than participants in Version Lab, as well as larger response time differences between congruent and incongruent pairings for participants in Version Lab compared to Version Web There was a decreasein mean standard error between Version Web (mean *SE* = 54) and Version Lab (mean *SE* = 64).

**Table 10:** The effect of the three-way interaction between Version, Stereotype, and Name Gender on response time. Table shows mean response time (ms), SD, and 95% confidence interval for each name/role pairing.

Version	Stereotype	Name Gender	Mean Response	SE	Lower Bound	Upper Bound
Version Web (PsyToolkit)	Feminine	Female	978	52	881	1087
		Male	1044	58	937	1164
	Non-Stereotyped	Female	954	57	848	1072
		Male	959	58	851	1081
	Masculine	Female	1001	60	890	1126
		Male	982	60	870	1107
Version Lab (E-Prime)	Feminine	Female	1135	61	1021	1262
		Male	1232	69	1105	1374
	Non-Stereotyped	Female	1112	67	988	1251
		Male	1132	70	1003	1278
	Masculine	Female	1154	70	1025	1300
		Male	1134	70	1005	1281

## Discussion

The aim of this study was to test the replicability of the Internet-based instrument PsyToolkit when compared to the laboratory-based implementation of E-Prime 3.0 for use with complex choice experiments through a psycholinguistic paradigm. Both PsyToolkit and E-Prime are psychological testing tools that are designed to be easy to use by students, having a low barrier to entry with coding requirements, and having extensive libraries of experiments. PsyToolkit was run on participants’ personal Internet-connected computers outside of laboratory conditions, while E-Prime was run on a single air-gated computer inside of laboratory conditions. The results of this study supported a high level of replicability between PsyToolkit and E-Prime, with Bayes factors indicating that we can accept the null hypothesis of no difference between Versions for Response. A secondary aim of this study was to examine the possibility that Internet-based experimentation might have a higher level of ecological validity than laboratory-based experimentation. It is possible that the ability to undertake experiments in familiar surroundings, and in the absence of researchers, could lead to participants being more comfortable while responding. If so, it would be expected that participants would be less affected by, for example, social desirability bias, meaning that their results should be more in line with how they would react in a naturalistic setting. If this was indeed the case, it would follow that participants who undertook the PsyToolkit version of the experiment would be more comfortable in responding negatively, and would overall respond more quickly, than those who undertook the E-Prime version of the experiment. The results of this study offer partial support for these assumptions, but this was to a very minor level, and as such cannot be generalised outside of this study.

Analyses in this study focused on both response choice and Response Time for positive responses. The automatic removal of Version from the models of best fit for both Response (at all levels) and Response Time (for two- and three-way interactions) indicates that there were no significant overall differences between the results obtained in PsyToolkit and E-Prime. Version was re-added at all levels to the final models that were analysed. The results for Response indicated that there were no significant main or interaction effects involving Version at the 95% confidence level, strongly supporting the idea that results obtained through PsyToolkit are in line with those obtained in a laboratory setting. The results for Response Time indicated that there were no interaction effects involving Version at the 95% confidence level, and, while a significant main effect of Version was found, this was found to indicate a general tendency towards faster responses for those who undertook the PsyToolkit version of the experiment, but overlapping 95% confidence intervals indicated that we cannot be sure that there is truly a difference in response time between responses received through PsyToolkit and E-Prime. As such, the results of Response Time also support the idea that results obtained through PsyToolkit are in line with those obtained in a laboratory setting.

Although no significant three-way interaction was found for either response or response time, some general variability between the two Versions can be seen. For Response, this variability takes the form of participants who undertook the PsyToolkit version of the experiment tending towards answering less positively, especially for female first name / masculine role nouns pairings, than those who undertook the E-Prime version of the experiment. For Response Time this takes the form of participants who undertook the PsyToolkit version tending to respond faster than those who undertook the PsyToolkit version, while participants who undertook the E-Prime version of the experiment had larger mean response time differences between congruent and incongruent pairings. While this is in line with the expected effects of ecological validity, it is also possible that the differences in mean Response Timefound between PsyToolkit and E-Prime are due to differences in the manner in which PsyToolkit and E-Prime measure reaction time. Further, the difference in Response Time standard errors between PsyToolkit and E-Prime (mean difference = 10) is in keeping with the concept of increased ecological validity.

It was expected that Response Time and standard deviations for both PsyToolkit and E-Prime should be higher than those found by Reimers and Stewart [15] and by Schubert et al. [16]. Mean Response Time in this study was found to be higher than mean Response Time for both Reimers and Stewart [15] (approximately 900ms) and for Experiment 5 of Schubert et al. [16] (approximately 800ms), and Response Time standard errors were higher than both Reimers and Stewart [15] and Schubert et al [16]. The increase in Response Time supports the idea that this is a more complex decision task than those used by Reimers and Stewart [15] and Schubert et al. [16].

Since data collection was completed, updates have been released for both PsyToolkit and for E-Prime 3.0. These updates have improved performance and may affect response time measurements for both PsyToolkit and E-Prime. However, as the results presented in this study already show high levels of replicability between the instruments, it is unlikely that these updates would remove this replicability, at least for the task at hand.

In conclusion, the results of the current study indicated that PsyToolkit is a viable method for conducting both general and psycholinguistic specific experiments that utilise CRT tasks, with effects found to replicate for both response choice and response time.
